# Superlubricity of Borophene: Tribological Properties in Comparison to hBN

**DOI:** 10.1021/acsnano.5c11587

**Published:** 2025-10-05

**Authors:** Antoine Hinaut, B. Sena Tömekçe, Shuyu Huang, Yiming Song, Ernst Meyer, Antonio Cammarata, Willi Auwärter, Thilo Glatzel

**Affiliations:** † Department of Physics, University of Basel, Klingelbergstrasse 82, 4056 Basel, Switzerland; ‡ Physics Department E20, TUM School of Natural Sciences, Technical University of Munich, 85748 Garching, Germany; § Department of Control Engineering, Faculty of Electrical Engineering, Czech Technical University in Prague, Technicka 2, 16627 Prague 6, Czech Republic

**Keywords:** borophene, hBN, nc-AFM, STM, 2D materials, superlubricity, friction

## Abstract

The tribological performance of 2D materials makes them good candidates toward a reduction of friction at the macroscale. Superlubricity has been observed for graphene, MoS_2_, and MXenes, whereas hexagonal boron nitride (hBN) is used to reduce or tune friction. Other materials are investigated as potential candidates for low-lubricity applications. Specifically, borophene is predicted to have ultralow friction. Here, we experimentally investigate the frictional properties of borophene and use a borophene/hBN lateral heterostructure to directly compare the tribological properties of the two complementary 2D materials. In particular, we investigate the friction between a sliding tip and (i) the weakly corrugated $X_{6}$ -borophene layer on Ir(111) or (ii) the hBN/Ir(111) superlattice structures with a strongly corrugated moiré reconstruction. Our experimental study performed in ultrahigh vacuum at room temperature combined with a Prandtl–Tomlinson (PT) model calculation confirms the superlubricity predicted for borophene, while hBN, which exhibits a higher friction, is nevertheless confirmed as a low friction material. Ab initio calculations show that the lower friction of $X_{6}$ -borophene with respect to hBN can be rationalized by weaker tip/surface interactions. In addition, we assess structural and electrical properties of borophene and hBN by using scanning probe techniques and compare their dissipation under the oscillating tip to investigate the possible path of energy dissipation occurring during friction. Our study demonstrates the low frictional properties of borophene and the potential of lateral heterostructure investigations to directly compare the properties of these 2D materials.

## Graphical abstract

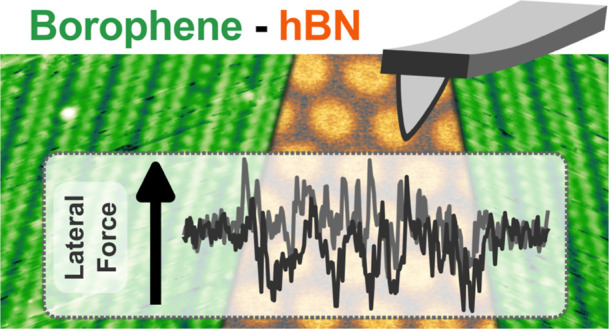

## Introduction

The tribological properties of 2D materials favor their use as solid lubricants.^1–4^ New materials that can reach a superlubric state are of interest for a variety of applications, particularly for reducing energy consumption. A superlubric state is a sliding regime where a near-zero friction is measured (i.e., a coefficient of friction near or below 0.001).^5–7^ Although selected 2D materials are already incorporated into devices, fundamental studies, down to the atomic scale, are needed to understand the origin of their low frictional properties.^8–14^ In particular, the atomic structures, electronic properties, and interactions with the support can have a major influence on the tribological performance. For monolayers, possible moiré structures are known to strongly influence the lubricity.^12,15^ Chemical modification, defects, or grain boundaries further influence lubricity.^16–18^ Furthermore, measurements of lateral heterostructures are of interest to directly compare the frictional behavior of 2D materials.^19,20^

Among 2D materials, borophene, a monolayer of boron atoms, has gained particular attention due to its interesting properties, such as electrical conductivity, mechanical strength, and chemical reactivity. First synthesized on an Ag(111) surface,^21,22^ borophenes by now have been prepared on various supports.^23–25^ As theoretically predicted and experimentally confirmed, different polymorphs can be formed, depending on the growth method, the support material, or other treatments that can influence the desired properties.^26–32^ The potential of borophene as tribological materials has been anticipated early on,^33^ and mechanical characteristics were modeled.^34–38^ Nonetheless, the respective experimental studies are largely missing. Specifically, outstanding tribological performances such as ultralow friction and even a negative friction coefficient are so far only theoretically predicted^39–42^ and yet to be proven experimentally. Hexagonal boron nitride (hBN), a stoichiometric mixture of boron and nitrogen atoms arranged in a honeycomb lattice, is established as a lubricant or as an intercalation layer, given its tribological performance.^10,43,44^ The formation of moiré patterns for surface-supported monolayer hBN adds interesting electronic and mechanical properties^45^ and allows hBN to be used as a nanotemplated support for atoms or molecules.^46^

Here, we grow lateral heterostructures to directly investigate and compare the structural, electrical, and tribological properties of borophene and hBN on Ir(111). The structures of the layers, e.g., $X_{6}$ -borophene/Ir­(111) and moiré formation for hBN/Ir(111), are probed by scanning tunneling microscopy (STM) and noncontact atomic force microscopy (nc-AFM) at room temperature in ultrahigh vacuum (UHV), complementing former low-temperature STM characterizations.^30^ We access the work function of both layers with Kelvin probe force microscopy (KPFM) as well as their dissipation in the presence of an oscillating tip with nc-AFM. Their tribological properties are then investigated via friction measurements using contact AFM in comparison with calculations using the Prandtl–Tomlinson (PT) model. The ultralow friction of the borophene layer is revealed experimentally for the first time. The hBN layer exhibits higher friction and remains wear-free. The better tribological performance of the borophene layer is rationalized by an atomistic model: while the tip/surface interaction is predicted to be weaker for $X_{6}$ -borophene compared to hBN, at the same time, the relatively higher friction of the hBN layer seems also to be related to the corrugated moiré pattern and its energy dissipation channel. Our results demonstrate the predicted interesting frictional properties of borophene and show the potential of lateral heterostructure measurements to directly compare 2D materials.

## Results and Discussion

### The Lateral Borophene/hBN Interface

#### Borophene and hBN Structures

Lateral heterostructures of borophene (denoted by B in all figures) and hBN are grown by dosing diborane and borazine onto a preheated (≃1200 K) clean and atomically flat Ir(111) surface maintained under UHV conditions, following a previously reported method^30^ (see Materials and Methods section for details). Borophene and hBN islands are obtained on the Ir(111) surface, with a total coverage close to a monolayer, as visible in the STM topography image in Figure 1a. Borophene and hBN domains with a size of more than hundred nanometers are formed. A hexagonal moiré structure is observed for the hBN domains.^45–48^ The borophene islands show a striped pattern characteristic for the $X_{6}$ polymorph on Ir(111), in agreement with studies at cryogenic temperatures.^28,30,49,50^ On a large scale, this is revealed as rows, indicated by the dotted lines in Figure 1a, showing two of the three possible rotational domains (120° between row orientation) on the surface. The borophene domains do not grow over Ir(111) step-edges, as indicated with the change in orientation when crossing the monoatomic Ir(111) step (following the dotted line). When formed on the same terrace, borophene and hBN have an apparent height difference of 35 pm at −0.3 V, which is in line with previous observations^30^ (see Figure S1 for height profiles).

**Figure 1:**
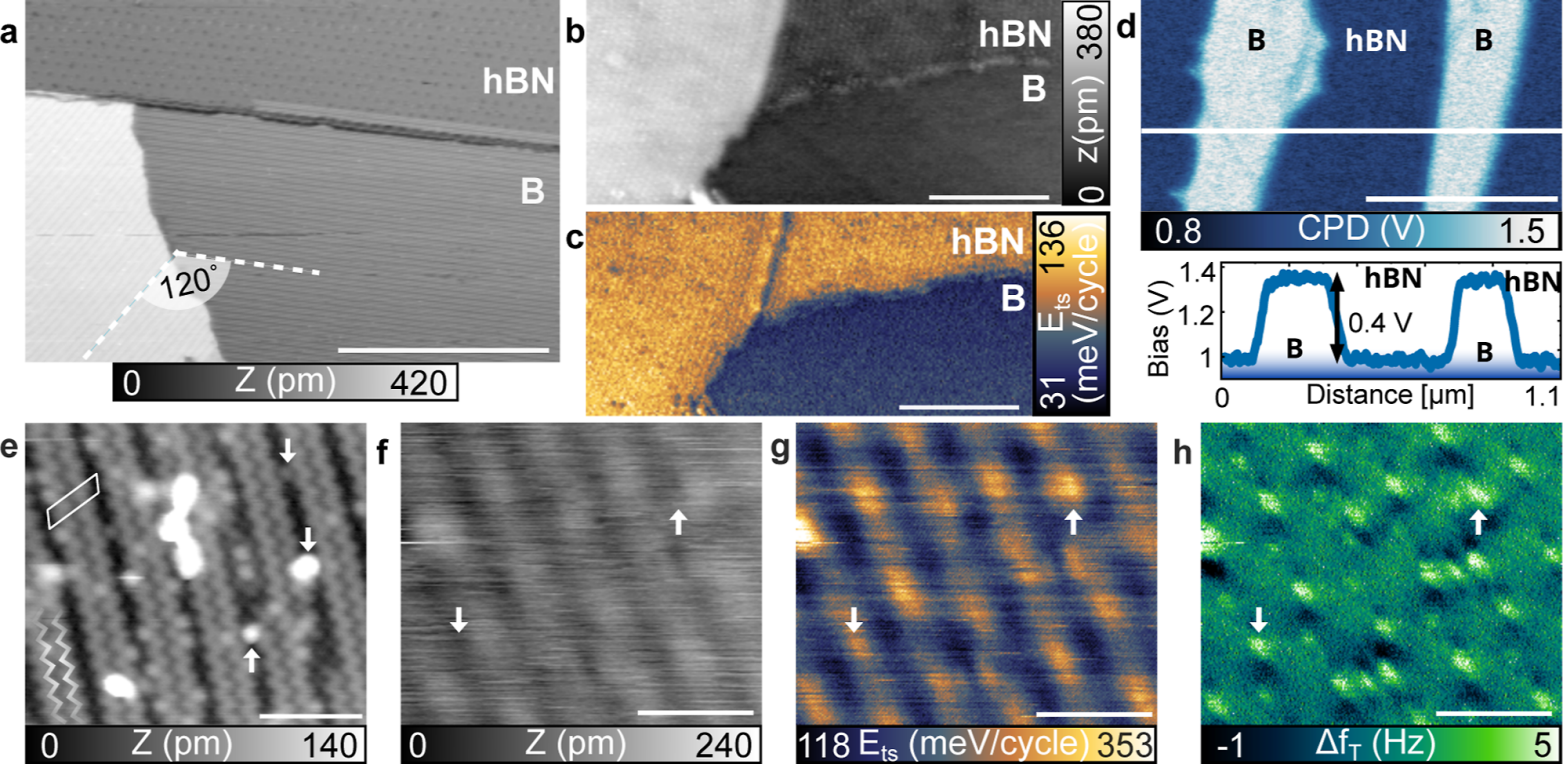
Large-scale borophene/hBN interface. (a) STM topography with a dotted line indicating borophene rows. (b) nc-AFM topography and corresponding (c) dissipation in meV per oscillation cycle. (d) CPD and corresponding profile. (e) High-resolution STM topography. (f) High-resolution nc-AFM topography and corresponding (g) dissipation (meV per oscillation cycle) and (h) torsional frequency shift. Parameters: (a) *I* = 200 pA, *U* = −0.3 V. (b,c) *f*_0_ = 164 kHz, *A* = 4 nm, Δ*f* = −17 Hz. (b,c) *f*_0_ = 164 kHz, *A* = 4 nm Δ*f* = −17 Hz. (d) *f*_1_ = 1.0682 MHz, *A* = 400 pm, Δ*f* = −20 Hz. (e) *I* = 90 pA, *U* = 1 V. (f,g,h) *f*_0_ = 169 kHz, *A* = 2 nm Δ*f* = −130 Hz, *f*_t_ = 1.53 MHz, *A*_t_ = 80 pm. Scale bars (a,b,c) 50 nm. (d) 500 nm. (e,f,g,h) 3 nm.

The large-scale lateral heterostructure of borophene/hBN on Ir(111) is also imaged by nc-AFM measurements; Figure 1b,c shows the topography and the simultaneously measured dissipation. The hBN islands are located on two Ir(111) terraces, where the lower terrace is shared by hBN and borophene. The hexagonal moiré structure of hBN is identified in both topography and dissipation. The monoatomic step for the Ir(111) surface below hBN shows a typical height (≈210 pm), whereas the hBN/borophene interface is nearly flat (see Figure S1 for details). The hBN layers separated by the Ir(111) step-edge exhibit identical dissipation, as is visible in the dissipation image of Figure 1c. The borophene layer shows a much lower dissipation, in meV per oscillation cycle, than the hBN. Such lower dissipation is a good indicator of the out-of-plane rigidity and adhesion of the borophene on the Ir(111) surface in comparison to the hBN on Ir(111).^45,51,52^ X-ray photoelectron spectroscopy (XPS) measurements have shown that borophene strongly interacts with Ir,^50^ with an interaction exceeding the one of hBN with Ir(111).^28,47^ The hBN layers are known to have a strong tendency to deform under an oscillating tip.^45^

#### Borophene Electronic Properties

We use KPFM in the frequency modulation mode to measure the contact potential difference (CPD) of the $X_{6}$ -borophene and hBN layers on Ir(111). Both borophene and hBN islands can be clearly distinguished in the CPD images, as shown in Figure 1d (see Figure S2 for corresponding topography and dissipation). The borophene island shows a higher CPD, corresponding to a higher work function than the hBN layer with a difference of 400 meV.^53^ Using the bare Ir(111) work function as a reference, we can estimate absolute work function values for hBN and borophene, see Figure S3 for more information. Assuming a work function of ≃5.78 eV for Ir(111),^27,54^ we evaluate the work functions of $X_{6}$ -borophene and hBN to be 4.68 and 4.28 eV, respectively. The value for borophene is close to the work function reported for a freestanding borophene sheet (4.75 eV) and different borophene polymorphs on Ag(111)^55^ but smaller than the one recently reported for borophene on Ir(111) with a potential influence of adsorbates (5.30 eV).^50^ The work function of hBN is also in good agreement with previous field emission resonance experiments (4.2–4.6 eV).^56^ The work function difference between borophene and hBN might have an effect on the electron dissipation channel and could influence the overall friction behavior.^57–59^

#### High-Resolution STM and AFM Measurements

The borophene $X_{6}$ reconstruction is more clearly identifiable in high-resolution STM images, as shown in Figure 1e. The stripped pattern is observed, and the characteristic “wavy” appearance of the rows is visible (see white overlay zigzag shape). The measured $X_{6}$ unit cell dimension of 1.65 nm × 0.60 nm with an internal angle of 60° matches literature values,^28,30,49^ see Figure S4 for dimension and profiles. Structural defects are observed with the appearance or disappearance of some rows as well as more local defects (indicated with white arrows).^30^ The details of hBN high resolution are shown in Figure S5.

The topography measured by nc-AFM on a $X_{6}$ -borophene island also reveals the presence of the rows, Figure 1f. Whereas in STM the “wavy” substructure of the rows is resolved, this is not the case in the nc-AFM measurements. Here, only the row structure is visible, with a measured width of 1.65 nm, see Figure S4 for a profile. The simultaneously measured dissipation (Figure 1g) and torsional frequency shift (Δ*f*_T_, Figure 1h) images also show the row structure of the borophene island. In the Δ*f*_T_ signal, the white protrusions observed between the rows (white arrows) are attributed to the defects and adsorbates that are also visible in the STM topography.^60,61^ In terms of dissipation, the lowest values are obtained on the areas identified as rows in the nc-AFM topography. The higher dissipation between the rows could be induced by the presence of the defects. A spatial analysis of the structures in the different images is provided in Figure S6.

The wavy pattern of the $X_{6}$ -borophene lattice, visible in our STM measurements, is mainly due to the electronic interaction at the borophene/Ir(111) interface.^28,49^ In nc-AFM, a technique more sensitive to the topography of the surface, less details of the electronic structures are observed, also when using torsional imaging.^13,62^ The lower dissipation of the borophene compared to hBN suggests a flatter and more rigid borophene layer compared to the more deformable moiré structure formed by hBN.^45,63^ This is also shown by calculating the dissipation ratio (Dr) between the highest and lowest dissipation over borophene and hBN layers, respectively. We found Dr_B_ ≃ 3 and Dr_hBN_ ≃ 5.4, indicating a larger variation over hBN than borophene, in good agreement with their deformability.

### Friction Properties of Borophene and hBN

#### Ultralow Friction of $X_{6}$ -Borophene

$X_{6}$ -Borophene and hBN domains are also distinguished by contact measurements, as shown in the forward lateral force image in Figure 2a. The $X_{6}$ -borophene is extended over two terraces in the upper part, while the hBN is detected in the lower part, as apparent from the moiré pattern. By changing the normal force, we performed load-dependent frictional force measurements on these borophene $\left(X_{6}\right)$ islands as shown in Figure 2b. The mean frictional forces are calculated by recording the forward and backward traces following the method described in ref 64. The $X_{6}$ -borophene exhibits superlubricity,^2^ as indicated by the calculated coefficient of friction of 1.2 × 10^–3^ for the lower loads. Such superlubric sliding is maintained up to a load value of 80 nN. For higher loads, the friction force becomes nonlinear and superlubricity disappears. However, by reduction of the normal force back below the threshold, the superlubricity can be reversibly restored. This is exemplified by the two measurements taken after the high loads (dark blue points in Figure 2b). The recovery of the low mean friction force value is an indication that both the tip and surface are preserved during the friction experiments (up to the maximum applied load).

**Figure 2:**
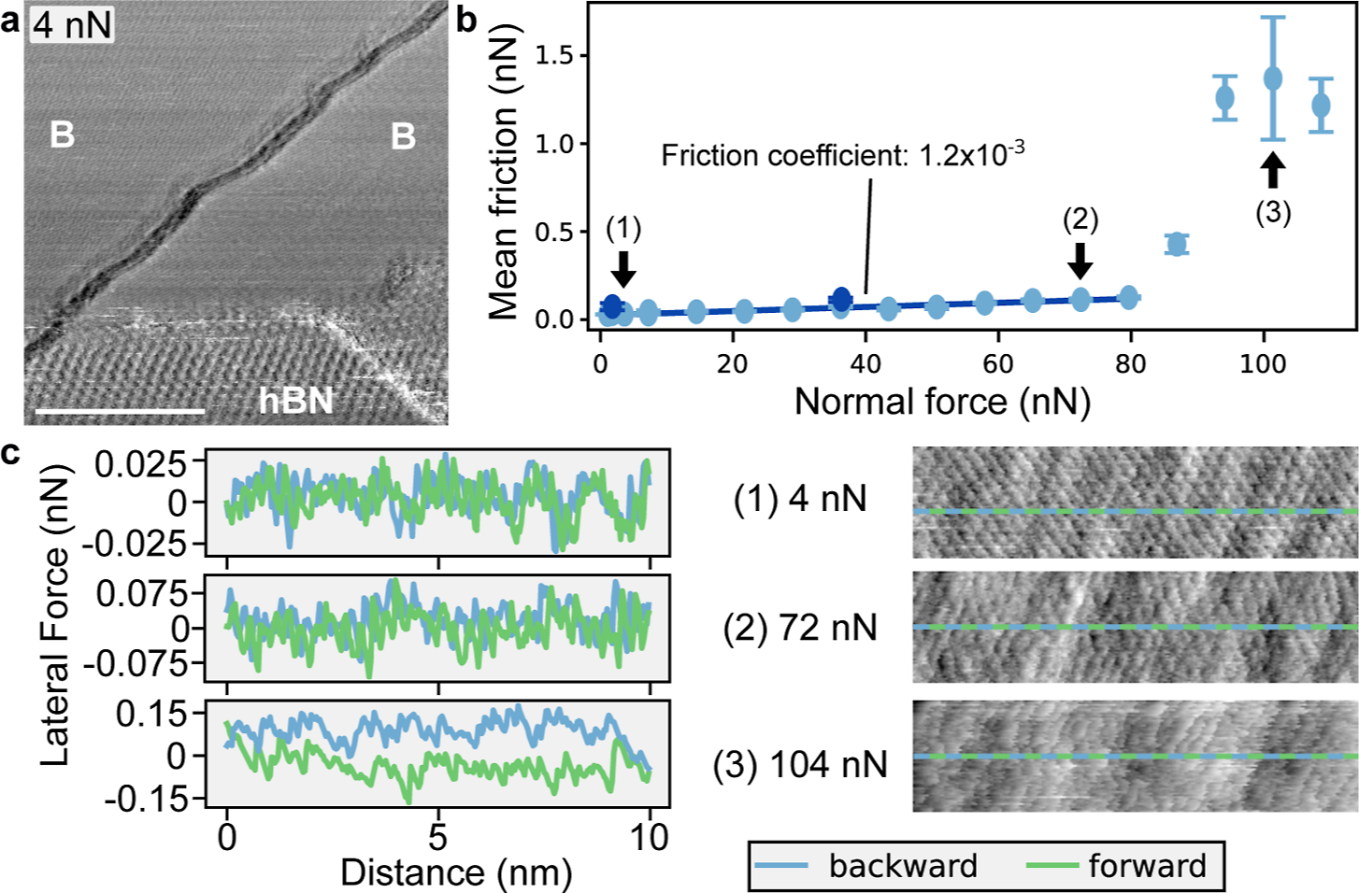
Borophene friction properties. (a) Lateral force map of the borophene hBN layers on Ir(111) at an applied load of 4 nN. (b) Mean friction force of the borophene layer. (c) Lateral force traces (left) and corresponding images (right) for applied loads of (1): 4, (2): 72, and (3): 104 nN. ν = 49 nm/s. Scale bar (a) 40 nm, images are 10 nm wide in (c).

The lateral force images and traces show the transition from the superlubric to the dissipative friction regime, as visible in Figure 2c, extracted from the mean friction measurement in Figure 2b (indicated by arrows). There, for low load and load just before transition (labels (1) and (2) on the graph), the forward and backward traces have no hysteresis. Atomic stick–slip is observed on borophene in the traces and atomic feature resolution is obtained in the lateral force images. At higher loads, without superlubricity (3 in the graph), a hysteresis is visible between the forward and backward traces. Furthermore, the atomic features are not observed in the lateral force image, indicating a frictional regime. The ultralow friction maintained over a wide range of normal loads, and its recovery after higher loads shows the excellent frictional properties of the borophene layer.

#### Friction of the Borophene/hBN Interface

A direct comparison of the frictional properties of both the borophene and hBN islands allows us to confirm the very low friction of borophene. To this end, we obtained the mean friction value on the hBN layer with a similar load dependency measurement, as shown in Figure 3a with the same tip. A friction coefficient of 2.0 × 10^–2^ is obtained for the hBN showing the much higher friction than borophene (1.2 × 10^–3^).

**Figure 3:**
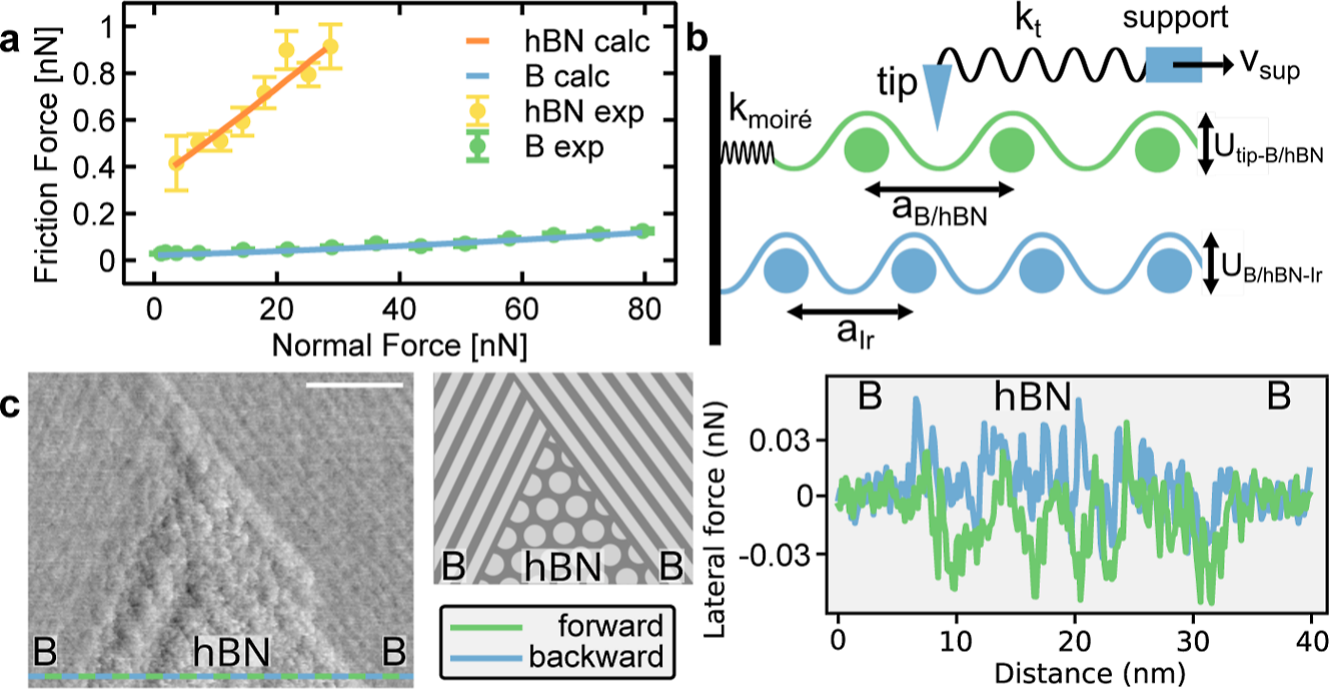
Friction properties of the (borophene/hBN)/Ir(111) lateral heterostructure. (a) Friction force versus load for borophene and hBN. Dots are from experiments, lines from PT model calculations. (b) Scheme of the PT model used for the calculations. (c) Lateral force image of the lateral borophene/hBN interface with the corresponding scheme and lateral force traces. Parameters: (c). Scale bar 10 nm.

The difference between hBN and $X_{6}$ -borophene in terms of their frictional behavior is also accessed by direct comparison on a contact trace, sliding over both hBN and $X_{6}$ -borophene under constant load. This is visible in the contact image and the corresponding scheme in Figure 3c, where an hBN island, embedded between two $X_{6}$ -borophene domains with different orientation, is scanned. The lateral force trace reveals the difference between hBN and the borophene. On $X_{6}$ -borophene islands, i.e., on the sides, forward and backward scans yield similar values and no hysteresis is observed, implying reduced friction (compare values in Figure 2). Above hBN, i.e., in the middle region, there is a hysteresis between forward and backward scans, indicating increased friction. The advantage of using a lateral heterostructure is clearly demonstrated here, with a direct comparison, in a single trace, revealing the higher friction of hBN compared to $X_{6}$ -borophene.

#### Prandtl–Tomlinson Model Applied at the Interface

To understand the difference in the lubricity regime between the two different 2D materials, we used a modified PT model^52,65^ on both borophene and hBN, including the effects of the moiré superstructure and its lateral flexibility. A scheme of the model and the parameters used is shown in Figure 3b. A tip is dragged at constant velocity by a spring over a sinusoidal potential, representing the interaction between the tip and the borophene or hBN layers. The underlying Ir(111), with a different lattice constant, naturally gives rise to a moiré superlattice. The lateral deformability of the moiré interface during sliding is characterized by a spring, *k*_moire_. The friction is determined by the product of the tip spring constant *k*_t_ and its tension between the tip apex position *x*_tip_ and the support position ν_s_ *t*. The potentials, with amplitude *U*_s_ and periodicity *a*_s_, are used to model the interaction between the tip and the 2D layers, where the subscript “s” (denoting substrate) should be specifically substituted with the corresponding material-specific parameters when describing borophene and hBN. We used *a*_B_ = 1.63 Å, *a*_hBN_ = 2.49 Å, and *a*_Ir_ = 2.71 Å as lattice dimensions extracted from our experiments and adjusted the values of *U*_B_ and *U*_hBN_, which represent the corrugation of the tip–surface interactions, to fit the calculations to the experimental values. Further details of the calculation can be found in the Supporting Information, part 6. To fit our calculations to the experimental values, we used a lower value for *U*_B_ than for *U*_hBN_. The reduced surface corrugation for borophene compared to hBN corresponds to a weaker energy barrier at the same normal force, which is in good agreement with experimental values. Specifically, *U*_hBN_ > *U*_B_ corresponds to the experimental finding that hBN has a higher friction than borophene for identical loads.

#### Potential Energy Surface of Borophene and hBN

The experimental observation as well as the predictions of the PT model and optimization with *U*_hBN_ > *U*_B_ are supported by our ab initio calculations. The strength of the tip–surface interaction has been evaluated by sampling the potential energy surface (PES) as a function of the position of the tip with respect to the surface. The PES scan has been realized by considering the model geometries for the $X_{6}$ -borophene and hBN system as in Figure 4. A 10 × 10 grid sampling of the PES was obtained by shifting the tip parallel to the (***a***, ***b***) plane with respect to the borophene or the hBN surface. We find that the potential energy barrier for the tip sliding over the surface is 0.03 and 0.12 eV/atom for the borophene and hBN systems, respectively (see Figure 4c). This points out that it is easier to slide over borophene than over hBN.

**Figure 4:**
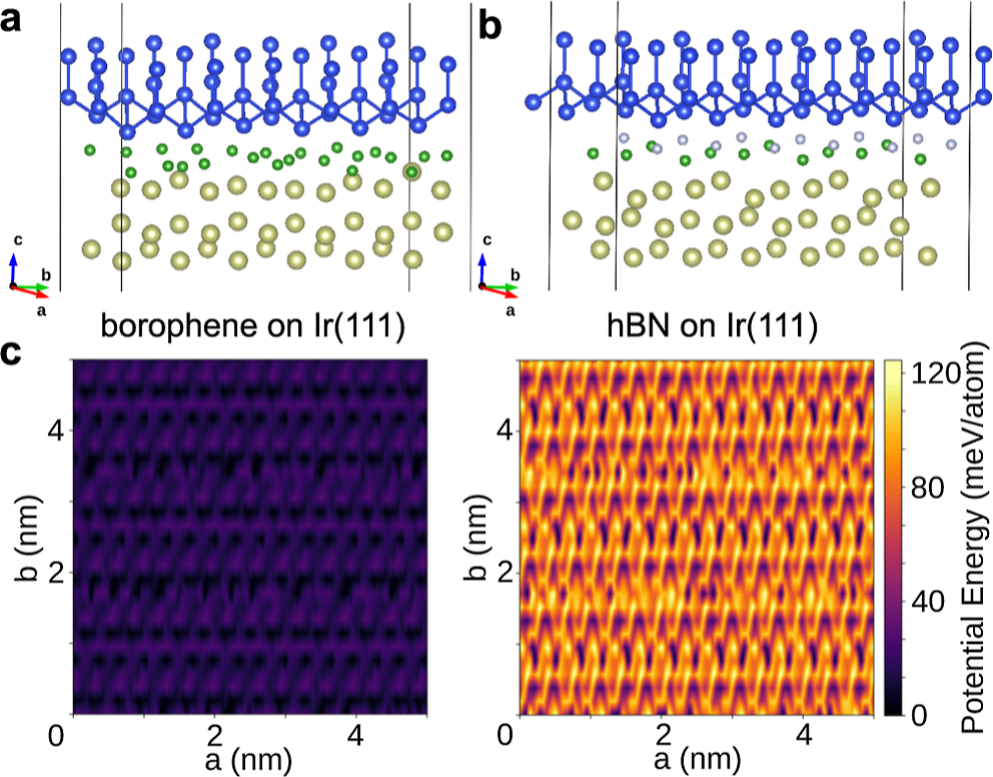
Schematic representation of the (a) Ir-borophene-tip and (b) Ir-hBN-tip geometric models used in the ab initio calculations. The golden, green, white, and blue spheres represent the position of the Ir, B, N, and Si atoms, respectively. (c) Potential energy surface (PES) thermal map of borophene on Ir(111) (left) and hBN on Ir(111) (right).

We also evaluated the vertical displacements of the B and N atoms in the two systems. We find that during sliding the B atoms in the borophene layer displace on average by 0.15 Å, while the atoms in the hBN layer displace by 0.43 Å. These results show that during sliding the borophene surface retains its flatness and has a weak interaction with the tip, while the hBN surface is more prone to deformations and has a strong interaction with the tip. Such results are in agreement with our STM–AFM measurements, as discussed above.

## Conclusion

We experimentally demonstrated the superlubricity of borophene for the first time. We took advantage of the possibility to create lateral heterostructures of borophene and hBN to directly compare their mechanical, electronic, and tribological properties. Using STM and nc-AFM at room temperature under UHV, we first characterized the borophene and hBN layers on the Ir(111) surface. We then measured a lower friction over borophene compared to hBN, which was further confirmed by both the PT model and ab initio calculations, showing a lower energy barrier for borophene, i.e., a lower friction force. The ab initio calculation also revealed a much higher vertical displacement of the hBN layer compared to borophene, which can be compared to the much higher friction measured experimentally. Our results demonstrate the potential of lateral heterostructure measurements to directly compare 2D material properties.

## Materials and Methods

### Borophene and Borophene/hBN Heterostructure Growth

The Ir(111) single crystals were prepared by repeated cycles of sputtering (Ar^+^ ions at an energy of 1 keV) and annealing at 1250 K. Borophene and borophene/hBN heterostructures were grown on Ir(111) by dosing diborane and/or borazine while keeping the substrates at high temperature.^30^ To promote the growth of the borophene/hBN lateral heterostructures, we dosed a diborane–borazine mixture, controlled by mass spectrometry, on a sample kept at 1200 K. To promote borophene, we dosed a diborane–borazine mixture onto the sample at 1350 K, resulting in the formation of pure borophene.^66^ The annealing is maintained for 10 min after the end of the dosing.

### Scanning Probe Experiments

STM, nc-AFM, and contact AFM measurements were performed with a home-built microscope operated at room temperature and controlled with Nanonis RC5 electronics. PPP-NCL cantilevers (Nanosensors) were used as sensors for nc-AFM (typical resonance frequencies of *f*_o_ = 160 kHz, *f*_1_ = 1 MHz, and *f*_t_ = 1.5 MHz, oscillation amplitude 2–5 nm, 400–800 pm, and 80 pm, respectively). PPP-CONT cantilevers (Nanosensors) were used for friction measurements. Cantilevers preparation consisted of an annealing for 1 h at 400 K followed by an Ar^+^ sputtering for 2 min at 1 keV at an Ar^+^ pressure of 3 × 10^–6^ mbar. STM tips were made from a Pt/Ir wire. The UHV system was maintained at a base pressure of 5 × 10^–11^ mbar during the measurements.

### PT Model Calculation

The dynamics of the friction system is described by the equation of motion:

1 $$\begin{array}{ll} m_{t}{ẋ}_{t}+m_{t}\mu_{t}\left({ẋ}_{t}-v_{s}\right) & =-\frac{\partial V\left(x_{t},t\right)}{\partial x_{t}};\\ m_{s}{ẋ}_{s}+m_{s}\mu_{s}{ẋ}_{s} & =-\frac{\partial V\left(x_{s},t\right)}{\partial x_{s}}. \end{array}$$

where *m*_t_ and *m*_s_ represent the effective mass of the tip and the locally deformed borophene or hBN. The damping coefficients of the corresponding motion μ_t_ and μ_s_ are set to a critical damping. The potential energy of the system *V* includes four contributions: the interaction potential between the tip and borophene/hBN, *V*_t–s_; the substrate–Ir(111) interface potential, *V*_s–Ir_; the cantilever spring potential; and the elastic strain energy associated with the moiré lateral deformation. This can be expressed as

2 $$V=V_{t-s}+V_{s-Ir}+\frac{1}{2}k_{t}{\left(x_{t}-v_{s}t\right)}^{2}+\frac{1}{2}k_{moire}x_{s}^{2}$$

where the interaction potentials are explicitly defined as

3 $$V_{t-s}=U_{t-s}\;\cos\left(\frac{2\pi \left(x_{t}-x_{s}\right)}{a_{s}}\right)$$

4 $$V_{s-Ir}=U_{s-Ir}\;\cos\left(2\pi \left(\frac{x_{t}}{a_{Ir}}-\frac{x_{t}}{a_{s}}+\frac{x_{s}}{a_{s}}\right)\right)$$

The top potential *V*_t–s_ with a graphene lattice constant of *a*_s_ (either borophene or hBN) and an amplitude of *U*_t–s_ describes the tip–surface interaction, which is superimposed by a bottom potential *V*_s–Ir_ representing the s–Ir(111) interaction, with an Ir(111) periodicity of *a*_Ir_ and an amplitude of *U*_s–Ir_. The stiffness *k*_t_ denotes the effective lateral spring constant of the cantilever and *k*_moiré_ represents the effective stiffness of the local elastic deformation of the upper moiré superstructure. As the cantilever moves at a constant velocity of *v*_s_, the tip and the material supercell are displaced by *x*_t_ for the tip and *x*_s_ for the moiré in-plane deformation.

The equations of motion are numerically solved using the fourth-order Runge–Kutta algorithm. To investigate load-dependent friction behavior, the amplitude of the interfacial potential amplitude is varied to modulate the normal load. The average friction under the corresponding corrugated potential is obtained by averaging the instantaneous friction over a fixed number of cycles in order to match the experimental length, i.e., 82 cycles, 20 nm and 62 cycles, 10 nm for hBN and borophene, respectively. All of the parameters used are shown in the Supporting Information, part 6.

### Ab Initio Calculations

The starting point to build the computational models for the X 6-borophene and hBN systems is the atomic geometries reported in,^28,47^ respectively. In our settings, the Ir surface, the layer, and the Si tip are arranged in the (***a***, ***b***) plane, while a 25 Å vacuum slab has been added along the ***c*** direction orthogonal to the layer plane, in order to prevent interactions between periodically repeated images (Figure 4). We performed Density Functional Theory calculations,^67^ as implemented in the vasp software.^68,69^ To describe the atomic interactions, we choose the Perdew–Burke–Ernzerhof (PBE)^70^ and the vdW-DF2 ^8^ energy functionals for the borophene and hBN systems, respectively. The plane wave energy cutoff is set to 500 eV, and the irreducible Brillouin zone is sampled with a 3 × 1 × 1 Monkhorst–Pack mesh.^71^ Self-consistent field and geometric relaxation loops are considered converged within a tolerance of 10^–8^ eV and 0.01 eV/Å, respectively. The atom positions and the lattice parameters a and b of the as-built Ir/ $X_{6}$ -borophene/Si and Ir/hBN/Si models have been optimized and used as starting points for sampling the potential energy surface. Since the atomic positions of the tip are fully optimized, surface reconstruction is taken into account, and no dangling bonds are present. The PES scan has been realized by displacing the position of the Si atoms along the ***a*** and ***b*** axes with respect to the borophene and hBN layers and relaxing the position of all the atoms forming the Ir/borophene/Si and Ir/hBN/Si interfaces; regarding the Si atoms, only their position along the ***c*** axis has been optimized, in order to prevent full relaxation which would restore the tip position to the starting point.

## Supplementary Material



## Notes

Borophene-hBN lateral interface with STM and nc-AFM: height profiles and dissipation profiles; work function of borophene and hBN: KPFM images and CPD profiles for work function evaluation; borophene on Ir(111): additional STM images and profiles; hBN on Ir(111): additional nc-AFM images and profiles; rows and defects in borophene with nc-AFM: additional images for defects positioning; and PT-model calculation: parameters used for PT-model calculation (PDF)
