# Supplementary material for: Superlubricity of Borophene: Tribological Properties in Comparison to hBN
Source: ACS Nano. 2025 Oct 5;19(41):36536–43. doi: 10.1021/acsnano.5c11587 (PMC12548358; doi:10.1021/acsnano.5c11587)
Supplement: Supplementary file 1 [file nn5c11587_si_001.pdf]

Supplementary information for: Superlubricity of Borophene: Tribological Properties in Comparison to hBN

Antoine Hinaut,[*,†] Birce Sena Tömekçe,[‡] Shuyu Huang,[†] Yiming Song,[†] Ernst Meyer,[†] Antonio Cammarata,[*,¶] Willi Auwärter,[*,‡] and Thilo Glatzel[*,†]

†*Department of Physics, University of Basel, Klingelbergstrasse 82, 4056 Basel, Switzerland*

‡*Physics Department E20, TUM School of Natural Sciences, Technical University of Munich, 85748 Garching, Germany*

¶*Department of Control Engineering, Faculty of Electrical Engineering, Czech Technical University in Prague, Technicka 2, 16627 Prague 6, Czech Republic*

E-mail: antoine.hinaut@unibas.ch; cammaant@fel.cvut.cz; wau@tum.de; thilo.glatzel@unibas.ch

Part 1: borophene-hBN lateral interface with STM and nc-AFM

Using the STM topography over the lateral heterostructure Fig. S1a, the height of the Ir(111) step edge is measured to be 190 pm (below borophene). In nc-AFM a height of 210 pm (below hBN) is measured as visible in the topography of Fig. S5b. The difference in height between $\mathcal{X}_6$-borophene and hBN is measured to be 35 pm in STM and is flat with nc-AFM (Fig. S5a,b. The dissipation channel of nc-AFM experiment reveals a lower dissipation on the borophene islands than on the hBN (Fig. S5b).

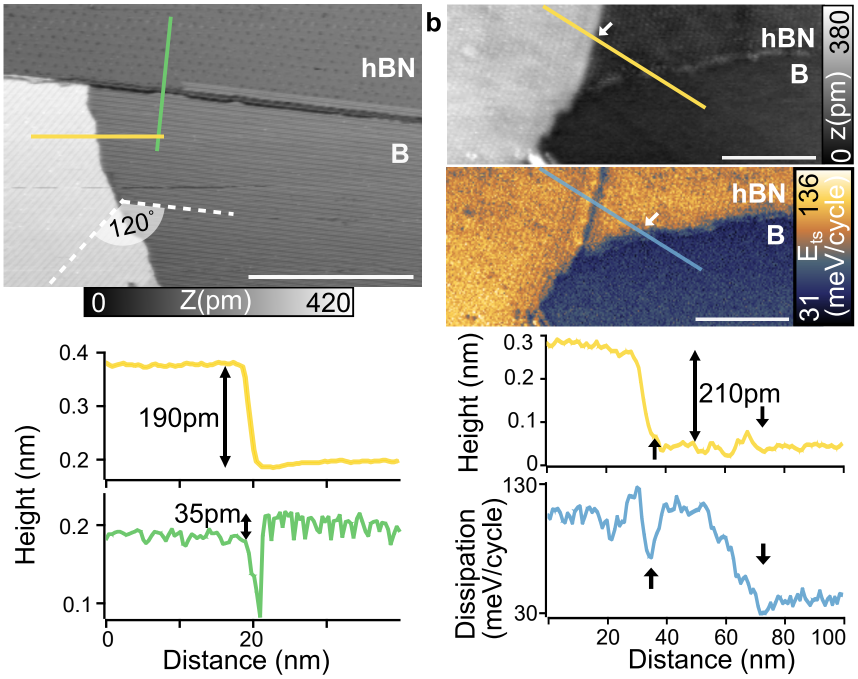

Figure S1: Borophene-hBN lateral interface on Ir(111) a) STM topography image and corresponding profiles. b) nc-AFM and corresponding dissipation images with profiles across lateral heterostructure. Arrows (black and white) are pointing to the step edge and the hBN-Borophene transition. Parameters: a) I = 200 pA, $U = -0.3$ V. b) f_0 = 164 kHz, $A = 4$ nm $\Delta f = -17$ Hz. Scale bars a,b) 50 nm

Part 2: Work function of Borophene and hBN

NcAFM topography, KPFM and dissipation images over borophene - hBN lateral interface. Larger area corresponding to Fig. 1d.

STM topography image over the $\mathcal{X}_6$-Borophene on Ir(111) surface. Showing both $\mathcal{X}_6$-borophene and Ir(111) area is shown in Fig. S3a. A zoomed STM topography revealing the $\mathcal{X}_6$-Borophene rows is visible in Fig. S3b. Using the Kelvin probe force image simultaneous to the large scale topography (Fig. S3a) and the corresponding profiles, as seen in Fig. S3c,d, we measure a contact potential difference of 1.1 V between $\mathcal{X}_6$-Borophene and Ir(111). Using the known work function for the Ir(111) surface ($\simeq$ 5.78 eV[1,2]), the value of the $\mathcal{X}_6$-Borophene work function on Ir(111) is calculated to be WF$_{(\mathcal{X}_6-Borophene)/Ir(111)}$ = 4.68 eV.

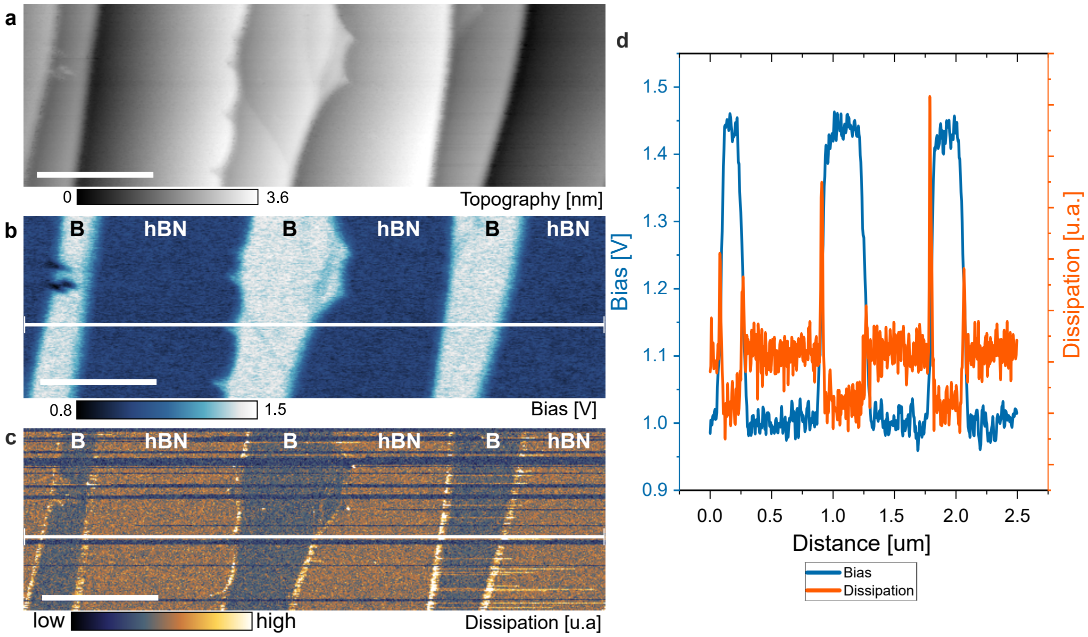

Figure S2: Borophene and hBN on Ir(111). a) NcAFM topography image. Larger area from Figure 1d of the main manuscript. b) Corresponding Bias (b) and Dissipation (c). Parameters: a-c) $f_0 = 10682$ kHz, $A = 400$ pm $\Delta f = -20$ Hz.

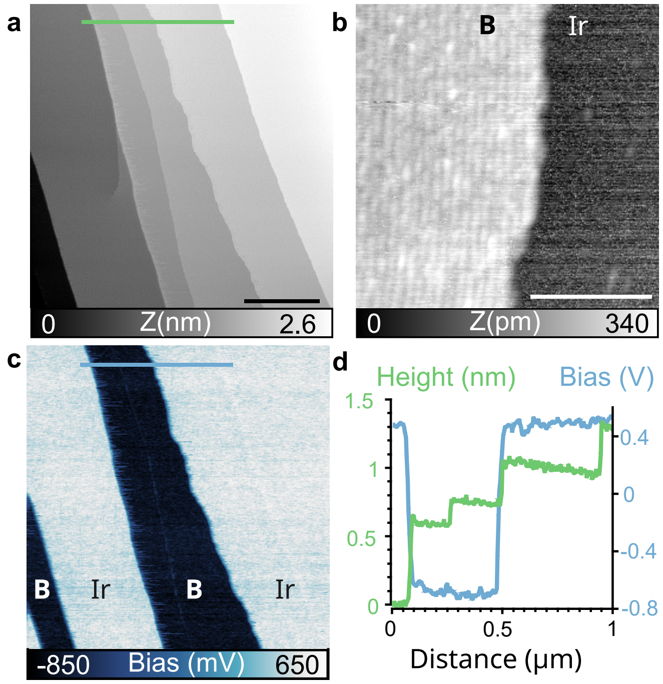

Figure S3: Borophene on Ir(111). a) NcAFM topography image of borophene grown on Ir(111). b) zoom topography image to reveal the $\mathcal{X}_6$-borophene reconstruction. c) CPD image simultaneous to a). Parameters: a-c) $f_0 = 169$ kHz, $A = 2$ nm $\Delta f = -130$ Hz.

Part 3: Borophene on Ir(111)

The dimensions of the $\mathcal{X}_6$-borophene are confirmed with profiles acquired on the STM and nc-AFM topography images as seen in Fig. S4. From STM (Fig. S4a), both directions of the $\mathcal{X}_6$ are obtained via profiles along and across the row structure while with nc-AFM (Fig. S4b), only the profile across the rows is possible. Corresponding profiles are visible in Fig. S4c. The $\mathcal{X}_6$ lattice unit cell measured dimension is 1.65 nm $\times$ 0.60 nm with an internal angle of 60° corresponding to the values reported in literature[3–5]

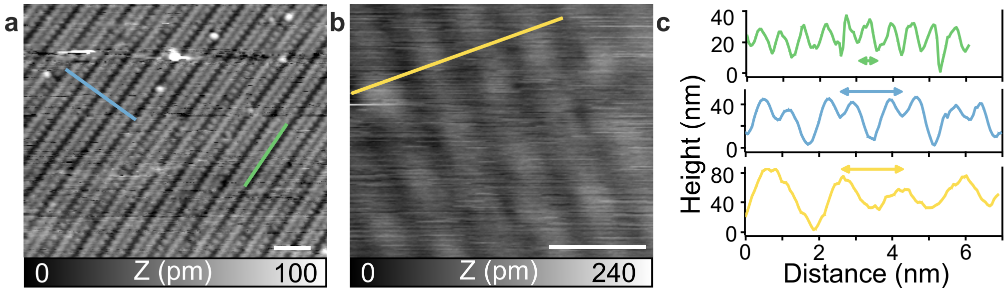

Figure S4: High resolution on $\mathcal{X}_6$ borophene-hBN. a) STM topography. b) nc-AFM topography. c) Profiles from a) and b). Parameters: a) $I = 250$ pA, $U = -1$ V; b) $f_0 = 169$ kHz, $A = 2$ nm, $\Delta f = -120$ Hz, Scale bars a,b) 3 nm.

Part 4: hBN on Ir(111)

When dosing pure borazine on Ir(111) on a sample maintained at 1200 K, a hBN monolayer is formed. Nc-AFM topography (Fig. S5a) and dissipation (Fig. S5b) images over the hBN reveal the extension of the layer overs hundreds of nanometers as well as the moiré pattern. Fig. S5c,d are better resolved topography and dissipation images. A lattice close to 2.9 nm is measured for the hBN moiré as reported in literature.[6,7] Moiré structure and lattice dimension of the hBN islands in the mixed sample as shown in Fig. 1 are identical to here.

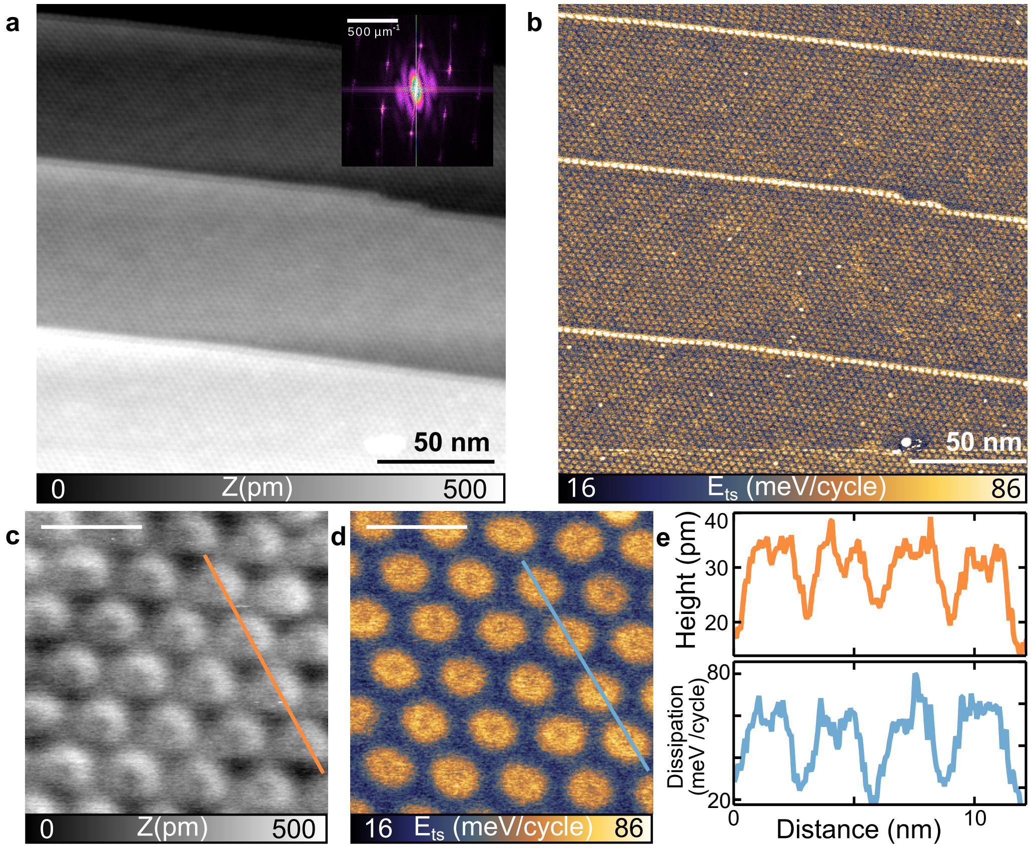

Figure S5: High resolution on hBN with nc-AFM. a) Large scale nc-AFM topography. Corresponding FFT in inset. b) Corresponding dissipation image in meV per oscillation cycle. c) Nc-AFM topography. b) Corresponding dissipation image in meV per oscillation cycle. e) Profiles from c) and d). Parameters: $f_0 = 169$ kHz, $A = 2$ nm, a,b) $\Delta f = -120$ Hz, c,d) $\Delta f = -130$ Hz. Scale bars a,b) 50 nm, inset 500 μm^{-1}, c,d) 5 nm.

Part 5: Rows and defects in borophene with nc-AFM

The rows structures and the defects in between them, as observed in nc-AFM topography and Δf_T images, are superimposed to other signals to reveal their influence in the various signals in the Fig. S6 and to allow correct identification of the rows structure of borophene in ncAFM. First, a mask (red) is created from the inter row lines observed in topography and then superimposed over the others signals (Fig. S6a). The same is done for the defects observed in Δf_T (Fig. S6b).

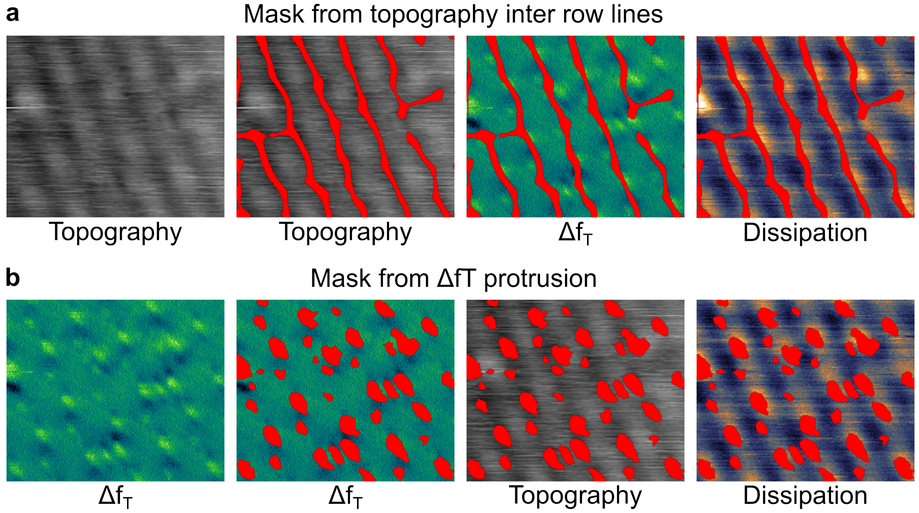

Figure S6: Rows and defects in borophene. a) Overlapping the mask from the inter rows lines from topography to Δf_T and dissipation. b) Overlapping the mask from the defects in Δf_T to topography and dissipation. $f_0 = 169$ kHz, $A = 2$ nm $\Delta f = -130$ Hz, $f_T = 1.53$ MHz, $A_T = 80$ pm. Image lateral size is 9 nm

The defects observed in the Δf_T signal are found in between the rows observed in the topography. This is similar to STM images, see Fig. 1e of the manuscript. Therefore the rows in nc-AFM coincide to the rows in STM.

Part 6: PT-Model calculation

The PT-model is used to fit mean friction from experiments.

Table 1: Parameters used for the calculation in the PT model and their respective extraction information

1	Torsional frequency of cantilever: 1551.6 kHz	from exp.
2	Lateral stiffness of cantilever ((k_t): 163.12937 N/m	from exp.
3	Stiffness of moiré ($k_{moiré}$): 163.12937 N/m	from[8]
4	Calculated mass of tip: 1.716×10^{-10} kg	from exp.
5	Mass of locally deformed borophene/hBN: 1.716×10^{-9} kg	
5	$a_{hBN} = 2.49$ Å	from exp
6	$a_{Ir} = 2.71$ Å	from exp
7	$a_B = 1.63$ Å	from exp
8	Damping coefficient of corresponding motions: $\eta_1 = 2 \cdot \sqrt{k_1/m_1} \cdot 1$ and $\eta_2 = 2 \cdot \sqrt{k_2/m_2} \cdot 1$	from[9]
9	Amplitude of corrugation potential: U_1 and U_2	best fit to exp
10	Temperature: 0 K	to simplify

Parameters are either extracted from experiments (indicated in such case), from literature or tunned to obtain for the best fitting between our calculation and our experiments.

The m_t is obtained from:

$$m_t = \frac{k_t}{(2\pi f_t)^2}$$

k_t is calibrated with common procedure[10] and f_t is obtained via a frequency sweep measurement. The mass m_s, of locally deformed borophene or hBN is set as 1/10 m_t and obtained from.[9] Lattices (a_{hBN},$a_{Ir(111)}$ and a_B) are extracted from topography of ncAFM experiments. Damping coefficient of corresponding motions are set to critical values as indicated in the Table 1. The temperature is set to 0K for simplification.

To obtain the amplitude corrugation potential, we manually adjusted the values to fit mean friction from experiments. The best fit results is shown in the Table 2 and plotted in the Fig. S7b.

Instantaneous friction obtained from the model is displayed in the Fig. S7a. The mean friction is calculated from averaging the points values in the instantaneous friction line. For

Table 2: Surface corrugation values used for plotting Fig. S7

Normal force	$U_{moiré\ hBN}$	U_{hBN}	Normal force	$U_{moiré\ B}$	U_{B}
(nN)	(*0.6eV)	(*0.6eV)	(nN)	(*0.6eV)	(*0.6eV)
3.6	6.50105	0.15004	1.086	1.00001	0.11
5.6	6.73973	0.158	3.086	1.00511	0.11242
7.6	6.97841	0.16596	5.086	1.01021	0.11484
9.6	7.21709	0.17392	7.086	1.01531	0.11726
11.6	7.45577	0.18188	15.086	1.03571	0.12694
13.6	7.69445	0.18984	21.086	1.05101	0.1342
15.6	7.93313	0.1978	29.086	1.07141	0.14388
17.6	8.17181	0.20576	37.086	1.09181	0.15356
19.6	8.41049	0.21372	43.086	1.10711	0.16082
21.6	8.64917	0.22168	51.086	1.12751	0.1705
23.6	8.88785	0.22964	57.086	1.14281	0.17776
25.6	9.12653	0.2376	65.086	1.16321	0.18744
27.6	9.36521	0.24556	73.086	1.18361	0.19712
			79.086	1.19891	0.20438

hBN, we averaged over 82 cycles to match our experimental scan length of 20 nm. For borophene, we averaged over 62 cycles to match our experimental scan length of 10 nm.

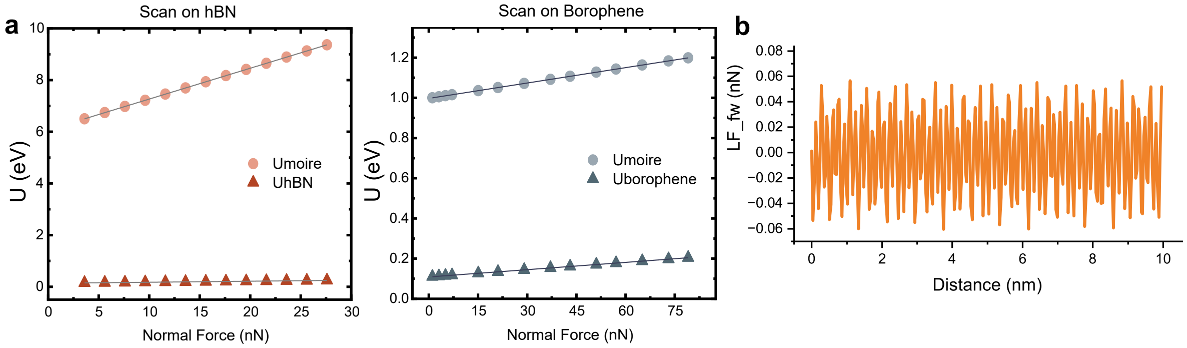

Figure S7: a) Relationship between surface corrugation and normal force for hBN (left) and Borophene (right). b) Line profile for the lateral force PT-model calculation.

References

(1) Derry, G. N.; Kern, M. E.; Worth, E. H. Recommended values of clean metal surface work functions. *Journal of Vacuum Science & Technology A* **2015**, *33*, 060801.

(2) Liu, X.; Wang, L.; Li, S.; Rahn, M. S.; Yakobson, B. I.; Hersam, M. C. Geometric

imaging of borophene polymorphs with functionalized probes. *Nature Communications* **2019**, *10*, 1642.

(3) Vinogradov, N. A.; Lyalin, A.; Taketsugu, T.; Vinogradov, A. S.; Preobrajenski, A. Single-Phase Borophene on Ir(111): Formation, Structure, and Decoupling from the Support. *ACS Nano* **2019**, *13*, 14511–14518.

(4) Cuxart, M. G.; Seufert, K.; Chesnyak, V.; Waqas, W. A.; Robert, A.; Bocquet, M.-L.; Duesberg, G. S.; Sachdev, H.; Auwärter, W. Borophenes made easy. *Science Advances* **2021**, *7*.

(5) Omambac, K. M.; Petrović, M.; Bampoulis, P.; Brand, C.; Kriegel, M. A.; Dreher, P.; Janoschka, D.; Hagemann, U.; Hartmann, N.; Valerius, P.; Michely, T.; Meyer zu Heringdorf, F. J.; Horn-von Hoegen, M. Segregation-Enhanced Epitaxy of Borophene on Ir(111) by Thermal Decomposition of Borazine. *ACS Nano* **2021**, *15*, 7421–7429.

(6) Farwick zum Hagen, F. H. et al. Structure and Growth of Hexagonal Boron Nitride on Ir(111). *ACS Nano* **2016**, *10*, 11012–11026.

(7) Auwärter, W. Hexagonal boron nitride monolayers on metal supports: Versatile templates for atoms, molecules and nanostructures. *Surface Science Reports* **2019**, *74*, 1–95.

(8) Huang, S.; Song, Y.; Hinaut, A.; Navarro-Marín, G.; Chen, Y.; Meyer, E.; Glatzel, T. Moiré Energy Dissipation Driven by Nonlinear Dynamics. *ACS Nano* **2025**, *19*, 17365–17373.

(9) Zhang, S.; Yao, Q.; Chen, L.; Jiang, C.; Ma, T.; Wang, H.; Feng, X.-Q.; Li, Q. Dual-Scale Stick-Slip Friction on Graphene/hBN Moiré Superlattice Structure. *Physical Review Letters* **2022**, *128*, 226101.

(10) Gnecco, E., Meyer, E., Eds. *Fundamentals of Friction and Wear on the Nanoscale*; NanoScience and Technology; Springer International Publishing: Cham, 2024.
